# Forecasted Dementia Prevalence in Portugal (2020-2080)

**DOI:** 10.1177/08919887241237220

**Published:** 2024-03-04

**Authors:** Sara Alves, Natália Duarte, Barbara Gomes

**Affiliations:** 1 Santa Casa da Misericórdia de Riba D’Ave/CIDIFAD – Centro de Investigação, Diagnóstico, Formação e Acompanhamento das Demências, Braga, Portugal; 2 CINTESIS@RISE, ICBAS, Porto, Portugal; 3 37830 Faculty of Medicine, University of Coimbra, Coimbra, Portugal

**Keywords:** dementia, community-dwelling, prevalence, population

## Abstract

Dementia is a global public health challenge, and its impact on Portugal is yet unclear. This study forecasts dementia prevalence in Portugal until 2080. Using the Gonçalves-Pereira et al (2021) method, we estimated dementia cases among older adults (≥65 years) in the community. Applying age-sex specific prevalence rates of the Gonçalves-Pereira study to population projections for Portugal between 2020-2080, based on the 10/66 Dementia Research Group criteria (10/66 DRG) and the Diagnostic and Statistical Manual of Mental Disorders IV criteria (DSM-IV), to Portugal’s population projections (2020-2080) under various growth scenarios (low, medium, and high). We anticipate a more than 2-fold increase in dementia prevalence from 2020 to 2080, both for 10/66 DRG [2.1%-5.0%] and DSM-IV [.8%–2.0%]. By 2080, those aged ≥80 years are projected to constitute 75.0% (vs 59.0% in 2020) of all dementia cases, particularly affecting women. Addressing dementia growth in Portugal calls for a comprehensive global response, while country-level estimates facilitate informed public health planning, policy-making, and resource allocation.

## Introduction

Dementia is a recognized public health concern with an impact on the people that live with the disease, their families, and caregivers. As population aging accelerates worldwide, the number of people living with dementia is set to rise rapidly in the decades to come. According to the World Health Organization,^1^ over 55 million people live with dementia worldwide and this number is estimated to increase to 139 million by 2050.

The available epidemiological studies identify substantial variations in the prevalence of dementia over time and across Europe,^2-7^ mostly related to methodological aspects (e.g. age groups, diagnostic criteria) or due to study locations (aged countries such as the South of Europe vs North of Europe; urban vs rural). However, epidemiological data on dementia are not available in many world regions due to the high cost and the complexity of conducting large-scale dementia screening studies, and low participation. These studies are crucial to understand dementia pathways, planning effective health and social care responses, and to anticipate and meet future care needs. In the absence of epidemiological evidence, realistic solutions are required, including the analysis of available national data to generate robust estimates of dementia prevalence.^8^

One recent study from G. B. D. Dementia Forecasting Collaborators^6^ analyzed the global burden of dementia and estimated a total of 57 million people living with dementia in 2019, with a projected increase to 153 million by 2050. Specifically, for Portugal, this study reported that 200 994 persons had dementia in 2019, with the number due to increase to 351 504 by 2050. Alzheimer Europe, in 2019,^3^ used raw data combined from studies of population-based regional samples^9-11^ to estimate that 193 516 people lived with dementia in Portugal in 2018, with the number expected to rise to 346 905 in 2050.

One of the first Portuguese studies on dementia prevalence was conducted in the north of the country,^9^ in 2 samples (urban and rural), using the Diagnostic and Statistical Manual of Mental Disorders IV (DSM-IV) criteria for dementia.^12^ The results found a prevalence of 2.7% (95% CI 1.9-3.8). Another study, conducted by Gonçalves-Pereira et al (2017)^11^ in the south of Portugal, also in 2 samples (urban and rural) of residents aged ≥65 years, revealed a prevalence of 9.5% (95% CI 7.8-10.9) using the 10/66 Dementia Research Group (10/66 DRG) criteria and of 3.7% (95% CI 3.0-5.0) using the DSM-IV criteria. A third study, conducted by Ruano et al, in 2019 in Oporto,^10^ reported a 1.3% estimate of dementia prevalence using the DSM-V criteria. Most recently, in 2021, Gonçalves-Pereira et al,^13^ based on their previous study (2017), estimated that 217 549 community dwellers individuals aged ≥65 years lived with dementia according to the 10/66 DRG criteria, from the total of 2 280 424 older adults (≥65 years) that resided in Portugal in 2019 (9.5%). The authors compared estimates using the DSM-IV criteria, which resulted in 85 162 individuals living with dementia (3.7% prevalence).

Considering such discrepancies in the prevalence of dementia between studies (1.3%–9.5%), the real number of people living with dementia in Portugal remains unclear. Notwithstanding, among the three studies, the study of Gonçalves-Pereira et al.,^11^ generates higher quality evidence according to the Alzheimer’s Disease International quality criteria for dementia prevalence studies,^13,14^ which include four criteria: sample size [.5 to 2 pts], study design [0 to 2 pts], response proportion [1 to 3 pts], diagnostic assessment [0 to 4 pts]. Concretely, it scores 10 out of 11 points contrasting with 6 out of 11 points, for Nunes et al.^9^ and Ruano et al.^10^ studies. The differences pertain to the study design [2 pts vs zero pts], response proportion [2 pts vs 1 and 2, respectively] and diagnostic assessment [4 pts vs 4 and 3, respectively]. In the sample size criteria, all studies scored equal [1 point].

Population aging worldwide, and particularly in Portugal, will likely determine an important rise in the number of people living with dementia as increased age is associated with the disease.^15-17^ This scenario poses several policy implications for the future, not only related to global aging policies but also age-related diseases, including dementia. Although the existing forecasts make it difficult to understand the future impact of the disease because they produced different estimates (e.g. different dementia criteria, different forecasts of population growth).^6^

In our study, we aimed to forecast the prevalence of dementia in Portugal between 2020-2080 by using different dementia criteria and population growth scenarios (low, medium, and high), according to age groups within the population aged ≥65 years, and calculating sex-specific estimates. From our knowledge, this is one of the first studies in Portugal that forecast dementia prevalence. These data are of utmost importance to support the implementation of a dementia national policy plan – which is still in development in Portugal – and to define effective strategies for dementia care globally.

## Materials and Methods

To estimate the number of people living with dementia in Portugal between 2020-2080, we started to understand the trajectory of the Portuguese population within this period, focusing our analyses on understanding its characteristics and trends. Specifically, we emphasized the older population (≥65 years), analyzing specific subgroups (i.e., 65-69 years, 70-74 years, 75-79 years, and 80+ years), given their higher risk of developing dementia. We extracted annual resident population estimates (2020-2080) from Statistics Portugal (National Statistical Institute), considering age- and sex-specific estimates under different population growth scenarios (low, medium, and high).^18^ These population growth scenarios account for different trends in fertility, mortality, and migration. The high growth scenario reflects an optimistic hypothesis; the medium growth scenario represents a central evolution hypothesis; and, the low scenario considered a pessimistic hypothesis.

After that, and aiming to forecast the number of Portuguese older adults with dementia living in the community between 2020-2080, we replicated the methodology used by Gonçalves-Pereira et al.^13^In this study, the authors employed dementia prevalence rates from their previous study^11^ to resident population estimates of 2019. Concretely, these dementia prevalence rates were established based on a sample of a Portuguese community sample aged ≥65 years “who were residents of urban and rural mapped catchment areas that reflected national typical scenarios” (p. 62).^11^ Using both 10/66 DRG criteria^11,14^ and DSM-IV criteria^12^ (see Table 1 for criteria details), the authors calculated the prevalence of dementia for urban and rural areas and for the overall of the two areas, stratifying by sex and age group (65-69 years, 70-74 years, 75-79 years, and 80+ years). Prevalence rates according to the 10/66 DRG were: for 65-69 years, .5% for women and 2.5% for men; for 70-74 years, 6.6% for women and 5.8% for men; for 75-79 years, it was 9.8% for women and 11.0% for men; and for the group of 80+ years, it was 19.6% for women and 18.4% for men. DSM-IV criteria rates for the same age groups and sexes were**:** for the .0% for women and .6% for men; 4.1% for women and 1.3% for men; 2.9% for women and 5.5% for men; it was 7.8% and 6.8%. The authors used a Poisson regression model to explore the effect of sociodemographic factors, such as age, sex, education level.

**Table 1.** Description of 10/66 DRG and DSM-IV Diagnostic Criteria for dementia.

10/66 DRG criteria	DSM-IV criteria
1) Information on the age and educational level, and household assets^a^	1) Memory impairment
2) The Geriatric Mental State (GMS), a semistructured clinical mental state interview that applies a computer algorithm (AGECAT), identifying organicity (probable dementia), depression, anxiety, and psychosis; also, the world health Organization Disability assessment Schedule 2.0	2) At least one of the following: a) Aphasia; b) apraxia; c) agnosia; d) disturbance in executive functioning
3) A cognitive module, including the community screening Instrument for dementia (CSI’D’) COGSCORE and the modified CERAD 10-word list learning task with delayed recall	3) The cognitive deficits in criteria 1) and 2) each cause significant impairment in social or occupational functioning and represent a significant decline from a previous level of functioning
4) A brief physical and neurological examination including the NEUROEX, a brief fully structured neurological assessment (ex. Objectified quantifiable measures of lateralizing signs, parkinsonism, ataxia, etc.)	4) The course is characterized by gradual onset and continuing cognitive decline
5) An informant interview, including the structured CSI-D informant Interview, RELSCORE scale, the modified history and Aetiology Schedule – Dementia diagnosis and Subtype, and Neuropsychiatric Inventory, assessing both the severity of the symptoms and related caregiver distress	5) The cognitive deficits in criteria 1 and 2 are not due to any of the following: a) Other central nervous system conditions that cause progressive deficits in memory and cognition; b) systemic conditions that are known to cause dementia; c) substance-induced conditions
	6) The cognitive deficits do not occur exclusively during the course of delirium
	7) The disturbance is not better accounted for by another Axis I disorder (e.g., major Depressive Episode, Schizophrenia)

^a^Obtained directly from the participant or the key informant when information was unreliable.

In our study, we aimed to estimate the numbers of the Portuguese older population living with dementia in the community under different population growth scenarios between 2020-2080 considering the prevalence rates from Gonçalves-Pereira et al.^13^ both in relation to 10/66 DRG and DSM-IV criteria and age- and sex-specific rates. This also involved estimating prevalence rates for the entire Portuguese population, with additional subgroup analyses based on age groups (≥65 years) and sex. Our analysis was conducted using Microsoft Excel (Office 16).

## Results

### Characteristics and Trends of the Portuguese Population Between 2020 to 2080

**Figure 1.**
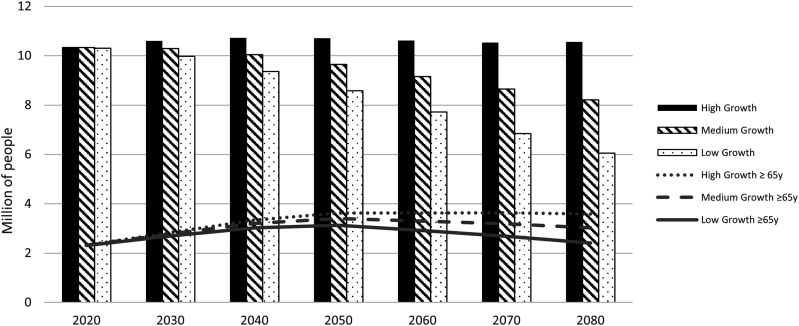
Estimated Portuguese resident population, displaying the share represented by people aged ≥65 years (lines), between 2020-2080 by population growth scenarios.

The population projections show a decrease in the total Portuguese resident population (in medium and low population growth scenarios) and the aging of the population for the next decades. In 2020, there were approximately 10.3 million Portuguese residents, of which 2.3 million were older adults (≥65 years), representing 22.5% of the national population (please see supplementary file 1 for more details about the prevalence by age- and sex-specific group from 2020-2080). For 2080, it is expected a decrease in the population in medium or low scenarios of population growth, respectively (Figure 1). For the high scenario of population growth, the Portuguese population will remain almost the same until 2080 (Figure 1). Despite this, older age groups (≥65 years) are predicted to increase substantially by 2050, representing at that time 33.9%, 35.3%, or 36.4% of the population in high, medium, or low growth scenarios of population growth, respectively. From 2050 onwards, older age groups will start to decrease but only in absolute numbers (please see supplementary file 1 for more details about the prevalence by age- and sex-specific group from 2020-2080). In relative terms, the proportion of older individuals in the population will continue to increase until 2070 for high and medium growth scenarios, or until 2080 for the low scenario of population growth. The group aged ≥80 years will face the greatest increase in comparison to the other older age groups, increasing 2.7 times between 2020 and 2080 in medium population growth scenario (compared to 1.5 times of increase of the aged 75-79 years, 1.2 times of the aged 70-74 years and .9 times of the aged 65-69 years).

Regarding sex population trends (please see supplementary file 1 for more details about the prevalence by age- and sex-specific group from 2020-2080), in 2020, men represented approximately 4.9 million of the national resident population (representing 47.1% of the total population). Until 2080, in medium and low growth scenarios, a decrease in the number of men is expected, with a similar decrease in women. From 2020 to 2080, a decrease in the proportion of men is predicted (47.1% in 2020 vs 45.0% in 2080 in the low growth scenario; and vs 46.1% in 2080 in the medium growth scenario), whereas the proportion of women is expected to increase (52.9% in 2020 vs 55.0% in 2080 in low growth scenario; and vs 53.9% in the medium scenario). In the high scenario, the proportion of men and women will remain similar between 2020 and 2080.

### Forecasted Prevalence of Dementia in Portugal Between 2020-2080

**Figure 2.**
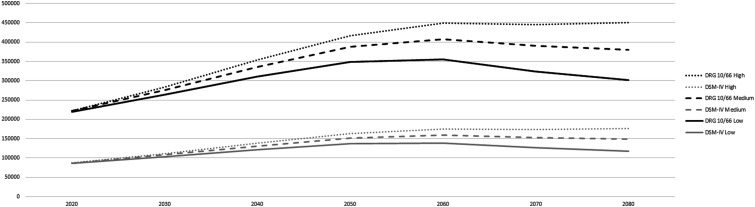
Estimates of dementia cases in Portugal between 2020 and 2080, by dementia diagnostic criteria and by population growth scenarios.

Applying age-sex specific dementia prevalence rates according to the 10/66 DRG and the DSM-IV criteria to people aged ≥65 years, we estimate the prevalence of dementia in the overall population to be, in 2020, 2.1% (n = 221 870) or .8% (n = 86 817) respectively (Figure 2). In 2080 and applying the 10/66 DRG criteria, the prevalence is estimated to rise to 4.3% (n = 450 715) in the high growth scenario, 4.6% (n = 380 603) in the medium, and 5.0% (n = 301 198) in the low growth scenario; applying the DSM-IV criteria, these prevalence rates are estimated to be 1.7% (n = 176 157), 1.8% (n = 148 916) or 2.0% (n = 117 983) (Figure 2). This means that from 2020 to 2080, the prevalence rate of people living with dementia in the community will increase 2-fold in high and medium-growth scenarios or 2.3 times in the low-growth scenario, considering both methods for dementia diagnosis.

#### Age-Specific Estimates

**Figure 3.**
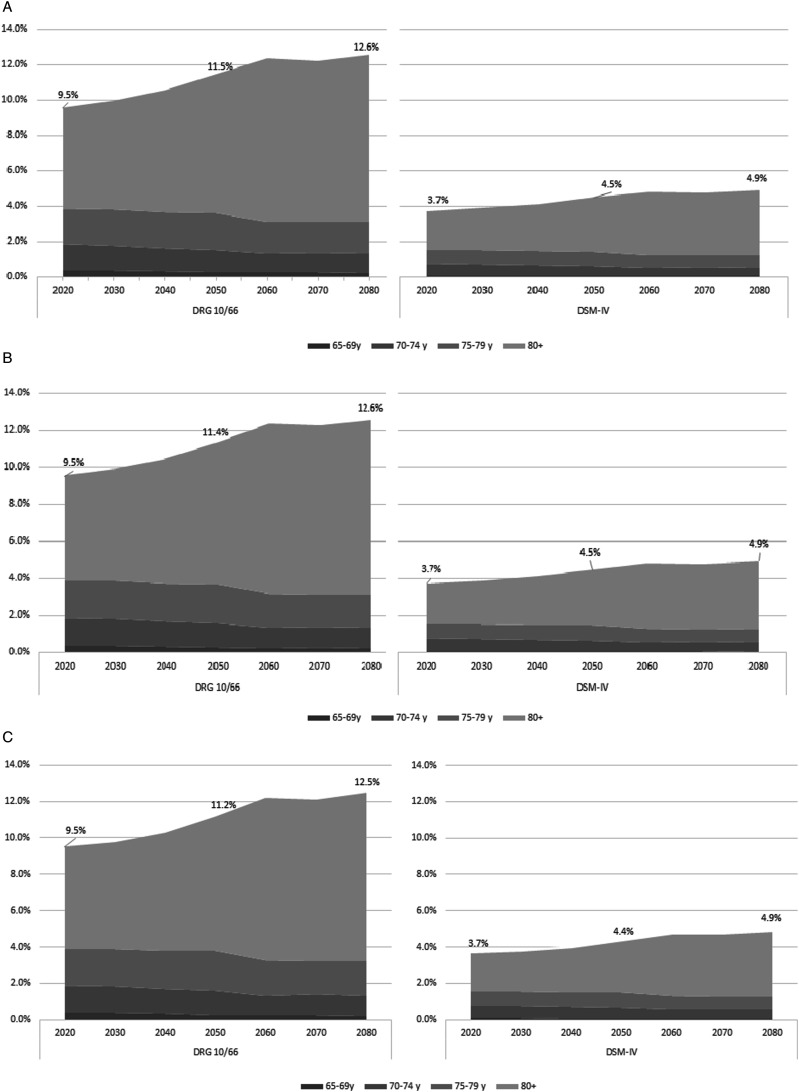
Estimates of dementia prevalence rates within adults aged ≥65 years between 2020-2080 by age group and dementia diagnostic criteria and population growth scenarios: (a) High growth scenario; (b) Medium growth scenario; (c) Low growth scenario.

In 2020, the prevalence rate of dementia among the older population (≥65 years) is estimated to be approximately 9.5% according to the DRG 10/66 criteria, and this is expected to rise to 12.6% until 2080 (Figure 3). Applying the DSM-IV criteria, the prevalence rate of dementia in the aged ≥65 years is estimated to be 3.7% in 2020, and expected to rise to 4.9% in 2080 (Figure 3). By 2080, people with dementia aged ≥80 years will represent nearly 75.0% of all cases of dementia, considering both 10/66 DRG and DSM-IV criteria and all scenarios of population growth (see in Supplementary File 1 the estimated number of dementia cases for every year from 2020 to 2080 by age group, sex, dementia diagnosis methods and population growth scenarios).

#### Sex-Specific Estimates

**Figure 4.**
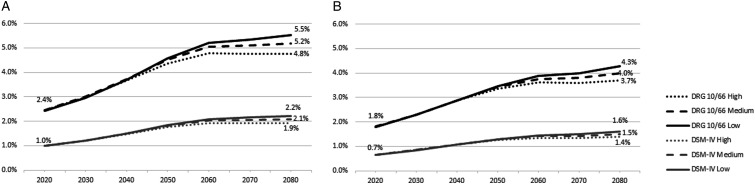
Sex-specific estimates of dementia prevalence rates for 2020-2080 by dementia diagnostic criteria and population growth scenarios: (a) Women; (b) Men.

When the subgroup of Portuguese women (approximately 5.5 million), in 2020, is considered, women with dementia represented 2.4% (n = 134 012) of all women living in Portugal, according to 10/66 DRG, and 1.0% (n = 54 635) according to DSM-IV (Figure 4). By 2080, the rate is expected to increase approximately 2 times to 4.8% (n = 265 414), 5.2% (n = 229 663), and 5.5% (n = 184 636) according to the 10/66 DRG criteria for high, medium, and low growth scenarios respectively; and to 1.9% (n = 106 703), 2.1% (n = 92 287), and 2.2% (n = 74 184) according to DSM-IV criteria (Figure 4).

Considering the subgroup of Portuguese men (approximately 4.9 million), in 2020, the prevalence rate of men with dementia was 1.8% (n = 87 809) according to 10/66 DRG, and .7% (n = 32 175) according to DSM-IV. The estimates for 2080 suggested these rates will increase approximately 2 times to 3.7% (n = 185 302), 4.0% (n = 150 938), and 4.3% (n = 116 562) according to the 10/66 DRG criteria for high, medium, and low growth scenarios respectively; and to 1.4% (n = 69 455), 1.5% (n = 56 629), and 1.6% (n = 43 799) according to DSM-IV criteria (Figure 4).

## Discussion

In this study, we forecasted the prevalence of dementia in Portugal between 2020-2080. This is the first-time data on dementia prevalence trends have been presented from this country. Existing studies show there is considerable uncertainty about the epidemiology of dementia, both nationally^9-11,13^ and internationally.^3,6^ These uncertainties are linked to variations in sample criteria (community vs institutionalized), age range, study design (one vs two phases design), cognitive battery assessment, sample size and location. Specifically, the study conducted by G. B. D. Dementia Forecasting Collaborators,^6^ which focused on relative risk-attributable prevalence associated with three dementia risk factors (high body-mass index, high fasting plasma glucose, and smoking) in people aged ≥40 years, estimated a total of 351 504 cases of dementia in Portugal by 2050. Our estimates using the 10/66 DRG criteria under a low growth scenario are not far (348 916 cases, see Supplementary File 1, Table C). Medium and high growth scenarios suggest the numbers may be even higher (up to 417 192 cases in the high growth scenario). On the other hand, using the DMS-IV criteria, the estimates are much lower (136 942, 151 964, or 163 474 cases in low, medium, or high growth scenarios, respectively).

The absence of a gold standard regarding the dementia diagnostic criteria as well as the underdiagnosis of dementia hampers more accurate projections. Considering our results, the two methods for dementia diagnosis evidenced very distinct estimates, which affect the understanding of the real magnitude of the problem. Having credible upper and lower bound estimates for the number of people living with dementia obtained with robust methods of diagnosis is therefore a priority for the planning of policies and services delivering health and social care, and resource allocation.

While the estimate of the number of potential cases of dementia in Portugal shows variation and uncertainty, it provides a basis for analyzing the extent to which diagnostic, post-diagnostic, and support services (e.g., training, awareness, social support) need to be developed to meet the needs of the potential number of people living with dementia in the country, their families and caregivers. The results presented in this study can help to anticipate the resources and plan ahead the support that will be required in the future. The difficulties regarding diagnosis, monitoring, treatment and care of people living with dementia already constitute key aspects today but are likely to become more important in the next decades. The expected increase in the prevalence rate of dementia in the Portuguese population (expected to more than double from 2020 to 2080, both for 10/66 DRG [2.1%-5.0%] and DSM-IV [.8%–2.0%]), reinforces the need to act immediately to fill these gaps. On one hand, this will contribute to improving the quality of life of patients and families, helping them to live better with the disease. On the other hand, it will aid countries to manage more efficiently the availability of resources in face of the need, and their associated costs.

Additionally, the high expected number of people living with dementia flags the need to consider end-of-life issues in patients in advanced stages of the disease, particularly the quality of the end-of-life care they receive, which impacts not only those who live with the disease but also their caregivers and family. Dementia is already the fourth cause of serious health-related suffering^19^ and the seventh leading cause of death worldwide .^1^ Bearing in mind the forecasts of dementia prevalence, dementia will probably rise in these rankings, meaning that a higher number of individuals could experience serious health-related suffering due to this disease in the future. Portugal and other countries in the world will therefore face growing needs for palliative care in dementia (e.g., specialized palliative care units, home-based palliative care teams, person-centered and evidenced-based palliative and supportive care interventions), which are currently very scarce in Portugal. Integrated care solutions that encompass all disease phases from diagnosis to palliative care are required to alleviate the suffering of people living with dementia as well their families and caregivers.

The estimated shrinkage of the Portuguese population for the next decades (under medium and low growth scenarios) as well as the rising number of persons aged ≥65 years – particularly those with ≥80 years – could challenge the response to dementia. The growth of older age groups increases the expected number of people that may suffer from dementia due to the relation of dementia and aging, requiring not only a higher number of services but also more specialized ones. In addition, dementia in advanced ages is frequently accompanied by the co-occurrence of chronic conditions (e.g., diabetes, depression, cardiovascular problems),^20^ which requires paying attention to aspects that are less frequent in younger people. Moreover, the changes in Portuguese demographics underline concerns regarding the number of (potentially available) caregivers (both formal and informal), which are likely to decrease substantially. Examining the caregiver support ratio^21,22^ – the number of potential caregivers aged 45-64 years (the most common caregiving age range) for each person aged ≥80 years (the subgroup that will increase most between 2020 and 2080 and at greater risk of having dementia) – the potential caregivers per older person were 4 in 2021 but are expected to be less than 1 in 2080. This aspect must, therefore, be taken into urgent consideration when planning future care. Specifically, countries should ponder dementia prevalence estimates to define a comprehensive dementia national plan that tracks and addresses caregivers’ needs over time.

This study also brought insights regarding sex-specific trends in the prevalence of dementia for the next decades. Our results show that dementia will remain more frequent in women than in men, with the former continuing to represent the majority of those living with dementia. This might be due to women’s longer life expectancy in comparison to men and the dementia risk factors attributable to sex as it seems to happen with APOE and total tau levels for Alzheimer’s disease.^23,24^ This information advises on the need to work closely with women by alerting them about the symptoms of the disease and through implementing prevention actions in this group.

Overall, our findings highlight the importance of considering future dementia prevalence estimates, given the huge impact this disease is expected to have. However, some limitations of the study are to be mentioned. Firstly, it focuses on dementia cases in people aged ≥65 years. Estimates of the number of those living with dementia could be higher if younger age groups were considered. Previous studies indicate that the percentage of dementia cases under 65 years is around .1%,^25^ predicted to be less than .5% in 2050.^6^ Therefore, the impact of this limitation on our estimated rates is likely minimal, but it requires further investigation because there is yet no Portuguese data on younger groups. Secondly, earlier manifestations of the disease such as mild cognitive impairment^26,27^ have not been considered. Prior research found a prevalence of mild cognitive impairment in 2.5% to 14.9% in people aged ≥60 years.^28^ This group deserves further separate attention. Thirdly, the methods of Gonçalves-Pereira et al.^11,13^ adopted by us to calculate the prevalence of dementia only consider community-dwellers individuals, excluding those institutionalized. Therefore, our findings only capture the prevalence of dementia in the community. Fourthly, modifiable risk factors may impact on future dementia prevalence trends, adding uncertainty to how trends will evolve in the future. Moreover, it is crucial to acknowledge that COVID-19 pandemic may impact the forecasts of future dementia cases. Infections have been associated with short-term cognitive decline.^29,30^ Further research is needed to understand their long-term effects, especially given the unprecedented number of people infected globally (including in Portugal). Fifth, we did not consider geographical variation within Portugal. In further studies, it would be interesting to explore potential disparities in dementia prevalence across regions in Portugal (e.g., urban vs rural; littoral vs interior) and regional projections, to inform local services planning. Sixth, we do not incorporate the influence of additional sociodemographic variables, such as educational level, into the forecasts of future dementia cases, which may exert some cohort effects. However, it is important to note that the prevalence rates used in this study, derived from the Gonçalves-Pereira study,^13^ have been previously adjusted for the impact of sociodemographic factors, including educational level. This adjustment is believed to mitigate potential biases associated with these factors. Seventh and lastly, it is important to understand the incidence of dementia as well as the stage and the severity of the disease to better plan services and resource allocation.

Despite limitations, the present study improves prior research about dementia in Portugal and worldwide^3,6,9-11,13^ by forecasting the prevalence of dementia over a 60-year period. From our knowledge, this is the first study in Portugal to forecast dementia prevalence trends considering different criteria for dementia diagnosis, age-sex-specific estimates, and population growth scenarios. The results are important for planning purposes and to help to inform health and social care authorities. Concretely, governments must be aware of the expected increase in dementia prevalence in the future to define a national strategy that addresses current and future challenges. The difficulties regarding the diagnosis of dementia (e.g., diagnostic criteria, late diagnosis) and the absence of current data on the national prevalence of dementia hamper more accurate forecasts of dementia and should be a priority for research. Investment is needed in high-quality epidemiological studies to have updated population-based information on trends of dementia over time, considering both its incidence and prevalence as well as geographical variation. Determining the level of investment needed in dementia care, including the detection of new cases, should be also considered as a future action.

## Conclusions

The results point out an expected increase in the prevalence of dementia in Portugal. Bearing in mind the burden that this disease has on patients, families, and society, it is crucial to clearly understand the magnitude of this disease. The estimated prevalence of dementia in Portugal in future years (according to 10/66 DRG criteria, it could reach 5% of the total Portuguese population in 2080), consubstantiates the importance of considering dementia care a public health priority and therefore, at the top of the list of major issues on national agendas. The increasing burden related to dementia suggests that a comprehensive approach to dementia is required, which includes defining a national plan considering prevention, early diagnosis, post-diagnosis support, long-term care and palliative care, specialized services, training, awareness, and research. Together these aspects will contribute to better living and dying with dementia, reducing avoidable suffering.

## Supplemental Material

Supplemental Material - Forecasted Dementia Prevalence in Portugal (2020-2080) Supplemental Material for Forecasted Dementia Prevalence in Portugal (2020-2080) by Sara Alves, Natália Duarte and Barbara Gomes in Journal of Geriatric Psychiatry and Neurology

## Data Availability

Data were extracted from official estimates on Nov 12, 2021, through the link: https://ine.pt/xportal/xmain?xpid=INE&xpgid=ine_indicadores&indOcorrCod=0010035&contexto=bd&selTab=tab2. The raw data supporting the conclusions of this article can be made available by the authors, upon reasonable request.
