# Supplementary material for: Forecasted Dementia Prevalence in Portugal (2020-2080)
Source: J Geriatr Psychiatry Neurol. 2024 Mar 4;37(5):403–12. doi: 10.1177/08919887241237220 (PMC11317018; doi:10.1177/08919887241237220)
Supplement: Supplemental Material - Forecasted Dementia Prevalence in Portugal (2020-2080) [file sj-pdf-1-jgp-10.1177_08919887241237220.pdf]

Supplementary file 1

Suppl. Table A – Estimated population and number of people with dementia in Portugal for 2020 - 2080 for the high growth scenario of population

| | **2020** | | | | | **2030** | | | | | **2040** | | | | | **2050** | | | | |
|---|---|---|---|---|---|---|---|---|---|---|---|---|---|---|---|---|---|---|---|---|
| | **65-69y** | **70-74y** | **75–79y** | **≥80 y** | **Total** | **65–69y** | **70–74y** | **75–79y** | **≥80 y** | **Total** | **65–69y** | **70–74y** | **75–79y** | **≥80 y** | **Total** | **65–69y** | **70–74y** | **75–79y** | **≥80 y** | **Total** |
| **Population**[a] | | | | | | | | | | | | | | | | | | | | |
| Women | 342328 | 309878 | 259585 | 439926 | **1351717** | 395024 | 357898 | 317567 | 569310 | **1639799** | 427093 | 387950 | 373578 | 742755 | **1931376** | 366480 | 409844 | 410329 | 919858 | **2106511** |
| Men | 288252 | 249657 | 188680 | 247284 | **973873** | 326683 | 290042 | 242497 | 340915 | **1200137** | 351037 | 311419 | 285478 | 462536 | **1410470** | 304205 | 329005 | 316304 | 575820 | **1525334** |
| Total | 630580 | 559535 | 448265 | 686611 | **2325590** | 721707 | 647940 | 560064 | 910225 | **2839936** | 778130 | 699369 | 659056 | 1205291 | **3341846** | 670685 | 738849 | 726633 | 1495678 | **3631845** |
| **Dementia cases according** | | | | | | | | | | | | | | | | | | | | |
| **10/66 DRG criteria** | | | | | | | | | | | | | | | | | | | | |
| Women | 1712 | 20514 | 25517 | 86269 | **134012** | 1975 | 23693 | 31217 | 111642 | **168527** | 2135 | 25682 | 36723 | 145654 | **210194** | 1832 | 27132 | 40335 | 180384 | **249683** |
| Men | 7293 | 14505 | 20830 | 45426 | **88054** | 8265 | 16851 | 26772 | 62626 | **114514** | 8881 | 18093 | 31517 | 84968 | **143459** | 7696 | 19115 | 34920 | 105778 | **167509** |
| Total | 9005 | 35019 | 46347 | 131695 | **222066** | 10240 | 40544 | 57989 | 174268 | **283041** | 11016 | 43775 | 68240 | 230622 | **353653** | 9528 | 46247 | 75255 | 286162 | **417192** |
| **DSM-IV criteria** | | | | | | | | | | | | | | | | | | | | |
| Women | 0 | 12643 | 7502 | 34490 | **54635** | 0 | 14602 | 9178 | 44634 | **68414** | 0 | 15828 | 10796 | 58232 | **84856** | 0 | 16722 | 11859 | 72117 | **100698** |
| Men | 1816 | 3221 | 10415 | 16815 | **32267** | 2058 | 3742 | 13386 | 23182 | **42368** | 2212 | 4017 | 15758 | 31452 | **53439** | 1916 | 4244 | 17460 | 39156 | **62776** |
| Total | 1816 | 15864 | 17917 | 51305 | **86902** | 2058 | 18344 | 22564 | 67816 | **110782** | 2212 | 19845 | 26554 | 89684 | **138295** | 1916 | 20966 | 29319 | 111273 | **163474** |

y = years; [a] Population estimates from 2020 to 2080 retrieved from INE (2021), https://ine.pt/xportal/xmain?xpid=INE&xpgid=ine_indicadores&indOcorrCod=0010035&contexto=bd&selTab=tab2

Suppl. Table A - Estimated population and number of people with dementia in Portugal for 2020 - 2080 for the high growth scenario of population (continuation)

| | | 2060 | | | | | 2070 | | | | | 2080 | | | | |
|---|---|---|---|---|---|---|---|---|---|---|---|---|---|---|---|---|
| | | 65-69y | 70-74y | 75–79y | ≥80 y | Total | 65–69y | 70–74y | 75–79y | ≥80 y | Total | 65–69y | 70–74y | 75–79y | ≥80 y | Total |
| **Population[a]** | | | | | | | | | | | | | | | | |
| | Women | 337264 | 331964 | 356593 | 1079145 | **2104966** | 343678 | 351810 | 331349 | 1064270 | **2091107** | 306940 | 330641 | 339814 | 1063679 | **2041074** |
| | Men | 288961 | 276851 | 280919 | 679788 | **1526519** | 302104 | 301351 | 272051 | 673378 | **1548884** | 268081 | 284758 | 288451 | 708384 | **1549674** |
| | Total | 626225 | 608815 | 637512 | 1758933 | **3631485** | 645782 | 653161 | 603400 | 1737648 | **3639991** | 575021 | 615399 | 628265 | 1772063 | **3590748** |
| **Dementia cases according** | **10/66 DRG criteria** | | | | | | | | | | | | | | | |
| | Women | 1686 | 21976 | 35053 | 211620 | **270335** | 1718 | 23290 | 32572 | 208703 | **266283** | 1535 | 21888 | 33404 | 208587 | **265414** |
| | Men | 7311 | 16085 | 31013 | 124877 | **179286** | 7643 | 17508 | 30034 | 123700 | **178885** | 6782 | 16544 | 31845 | 130130 | **185301** |
| | Total | 8997 | 38061 | 66066 | 336497 | **449621** | 9361 | 40798 | 62606 | 332403 | **445168** | 8317 | 38432 | 65249 | 338717 | **450715** |
| | **DSM-IV criteria** | | | | | | | | | | | | | | | |
| | Women | 0 | 13544 | 10306 | 84605 | **108455** | 0 | 14354 | 9576 | 83439 | **107369** | 0 | 13490 | 9821 | 83392 | **106703** |
| | Men | 1820 | 3571 | 15507 | 46226 | **67124** | 1903 | 3887 | 15017 | 45790 | **66597** | 1689 | 3673 | 15922 | 48170 | **69454** |
| | Total | 1820 | 17115 | 25813 | 130831 | **175579** | 1903 | 18241 | 24593 | 129229 | **173966** | 1689 | 17163 | 25743 | 131562 | **176157** |

y = years; [a] Population estimates from 2020 to 2080 retrieved from INE (2021), https://ine.pt/xportal/xmain?xpid=INE&xpgid=ine_indicadores&indOcorrCod=0010035&contexto=bd&selTab=tab2

Suppl. Table B - Estimated population and number of people with dementia in Portugal for 2020 - 2080 for the medium growth scenario of population

| | | **2020** | | | | | **2030** | | | | | **2040** | | | | | **2050** | | | | |
|---|---|---|---|---|---|---|---|---|---|---|---|---|---|---|---|---|---|---|---|---|---|
| | | **65-69y** | **70-74y** | **75–79y** | **≥80 y** | **Total** | **65–69y** | **70–74y** | **75–79y** | **≥80 y** | **Total** | **65–69y** | **70–74y** | **75–79y** | **≥80 y** | **Total** | **65–69y** | **70–74y** | **75–79y** | **≥80 y** | **Total** |
| **Population**[a] | | | | | | | | | | | | | | | | | | | | | |
| | Women | 342077 | 309558 | 259173 | 440331 | **1351139** | 391069 | 354000 | 312953 | 553566 | **1611588** | 418129 | 379258 | 363939 | 704429 | **1865755** | 351158 | 395700 | 395576 | 858632 | **2001066** |
| | Men | 287928 | 249308 | 188307 | 246329 | **971872** | 321435 | 284781 | 236752 | 325510 | **1168478** | 339008 | 299767 | 272933 | 424745 | **1336453** | 284748 | 310125 | 296700 | 513186 | **1404759** |
| | Total | 630005 | 558866 | 447480 | 686660 | **2323011** | 712504 | 638781 | 549705 | 879076 | **2780066** | 757137 | 679025 | 636872 | 1129174 | **3202208** | 635906 | 705825 | 692276 | 1371818 | **3405825** |
| **Dementia cases according** | **10/66 DRG criteria** | | | | | | | | | | | | | | | | | | | | |
| | Women | 1710 | 20524 | 25477 | 86349 | **134060** | 1955 | 23470 | 30763 | 108554 | **164742** | 2091 | 25145 | 35775 | 138139 | **201150** | 1756 | 26235 | 38885 | 168378 | **235254** |
| | Men | 7285 | 14485 | 20789 | 45251 | **87810** | 8132 | 16546 | 26137 | 59796 | **110611** | 8577 | 17416 | 30132 | 78026 | **134151** | 7204 | 18018 | 32756 | 94272 | **152250** |
| | Total | 8995 | 35009 | 46266 | 225698 | **221870** | 10087 | 40016 | 56900 | 168350 | **275353** | 10668 | 42561 | 65907 | 272903 | **335301** | 8960 | 44253 | 71641 | 262650 | **387504** |
| | **DSM-IV criteria** | | | | | | | | | | | | | | | | | | | | |
| | Women | 0 | 12630 | 7490 | 34522 | **54642** | 0 | 14443 | 9044 | 43400 | **66887** | 0 | 15474 | 10518 | 55227 | **81219** | 0 | 16145 | 11432 | 67317 | **94894** |
| | Men | 1814 | 3216 | 10395 | 16750 | **32175** | 2025 | 3674 | 13069 | 22135 | **40903** | 2136 | 3867 | 15066 | 28883 | **49952** | 1794 | 4001 | 16378 | 34897 | **57070** |
| | Total | 1814 | 15846 | 17885 | 88892 | **86817** | 2025 | 18117 | 22113 | 65535 | **107790** | 2136 | 19341 | 25584 | 106794 | **131171** | 1794 | 20146 | 27810 | 102214 | **151964** |

y = years; [a] Population estimates from 2020 to 2080 retrieved from INE (2021), https://ine.pt/xportal/xmain?xpid=INE&xpgid=ine_indicadores&indOcorrCod=0010035&contexto=bd&selTab=tab2

Suppl. Table B - Estimated population and number of people with dementia in Portugal for 2020 - 2080 for the medium growth scenario of population (continuation)

| | | 2060 | | | | | 2070 | | | | | 2080 | | | | |
|---|---|---|---|---|---|---|---|---|---|---|---|---|---|---|---|---|
| | | **65-69y** | **70-74y** | **75–79y** | **≥80 y** | **Total** | **65–69y** | **70–74y** | **75–79y** | **≥80 y** | **Total** | **65–69y** | **70–74y** | **75–79y** | **≥80 y** | **Total** |
| **Population[a]** | | | | | | | | | | | | | | | | |
| | Women | 309483 | 309732 | 336781 | 993764 | **1949760** | 302431 | 314910 | 300211 | 954321 | **1871873** | 258765 | 284216 | 295821 | 920176 | **1758978** |
| | Men | 257578 | 249989 | 255627 | 591780 | **1354974** | 258186 | 310125 | 236335 | 565024 | **1369670** | 218087 | 235778 | 240950 | 572244 | **1267059** |
| | Total | 567061 | 559721 | 592408 | 1585544 | **3304734** | 560617 | 625035 | 536546 | 1519345 | **3241543** | 476852 | 519994 | 536771 | 1492420 | **3026037** |
| **Dementia cases according** | **10/66 DRG criteria** | | | | | | | | | | | | | | | |
| | Women | 1547 | 20535 | 33106 | 194877 | **250065** | 1512 | 20879 | 29511 | 187142 | **239044** | 1294 | 18844 | 29079 | 180447 | **229664** |
| | Men | 6517 | 14524 | 28221 | 108710 | **157972** | 6532 | 15152 | 26091 | 103795 | **151570** | 5518 | 13699 | 26601 | 105121 | **150939** |
| | Total | 8064 | 35059 | 61327 | 246849 | **408037** | 8044 | 38897 | 55602 | 290937 | **390614** | 6812 | 32543 | 55680 | 191470 | **380603** |
| | **DSM-IV criteria** | | | | | | | | | | | | | | | |
| | Women | 0 | 12637 | 9733 | 77911 | **100281** | 0 | 12848 | 8676 | 74819 | **96343** | 0 | 11596 | 8549 | 72142 | **92287** |
| | Men | 1623 | 3225 | 14111 | 40241 | **59200** | 1627 | 3364 | 13046 | 38422 | **56459** | 1374 | 3042 | 13300 | 38913 | **56629** |
| | Total | 1623 | 15862 | 23844 | 95468 | **159481** | 1627 | 16849 | 21722 | 113241 | **152802** | 1374 | 14638 | 21849 | 73435 | **148916** |

y = years; [a] Population estimates from 2020 to 2080 retrieved from INE (2021), https://ine.pt/xportal/xmain?xpid=INE&xpgid=ine_indicadores&indOcorrCod=0010035&contexto=bd&selTab=tab2

Suppl. Table C - Estimated population and number of people with dementia in Portugal for 2020 - 2080 for the low growth scenario of population

| | | 2020 | | | | | 2030 | | | | | 2040 | | | | | 2050 | | | | |
|---|---|---|---|---|---|---|---|---|---|---|---|---|---|---|---|---|---|---|---|---|---|
| | | **65-69y** | **70-74y** | **75–79y** | **≥80 y** | **Total** | **65–69y** | **70–74y** | **75–79y** | **≥80 y** | **Total** | **65–69y** | **70–74y** | **75–79y** | **≥80 y** | **Total** | **65–69y** | **70–74y** | **75–79y** | **≥80 y** | **Total** |
| **Population**[a] | | | | | | | | | | | | | | | | | | | | | |
| | Women | 341828 | 309254 | 258775 | 434001 | **1343858** | 386817 | 349882 | 308062 | 519502 | **1564263** | 407612 | 369519 | 353321 | 640092 | **1770544** | 332902 | 378901 | 378440 | 759848 | **1850091** |
| | Men | 287663 | 249022 | 188008 | 243762 | **968455** | 316238 | 279569 | 231059 | 307580 | **1134446** | 326858 | 287902 | 260029 | 383641 | **1258430** | 265223 | 290878 | 276412 | 445480 | **1277993** |
| | Total | 629491 | 558276 | 446783 | 677763 | **2312313** | 703055 | 629451 | 539121 | 827082 | **2698709** | 734470 | 657421 | 613350 | 1023733 | **3028974** | 598125 | 669779 | 654852 | 1205328 | **3128084** |
| **Dementia cases according** | **10/66 DRG criteria** | | | | | | | | | | | | | | | | | | | | |
| | Women | 1709 | 20473 | 25438 | 85108 | **132728** | 1934 | 23162 | 30282 | 101874 | **157252** | 2038 | 24462 | 34731 | 125522 | **186753** | 1665 | 25083 | 37201 | 149006 | **212955** |
| | Men | 7278 | 14468 | 20756 | 44779 | **87281** | 8001 | 16243 | 25509 | 56502 | **106255** | 8270 | 16727 | 28707 | 70475 | **124179** | 6710 | 16900 | 30516 | 81835 | **135961** |
| | Total | 8987 | 34941 | 46194 | 129887 | **220009** | 9935 | 39405 | 55791 | 158376 | **263507** | 10308 | 41189 | 63438 | 195997 | **310932** | 8375 | 41983 | 67717 | 230841 | **348916** |
| | **DSM-IV criteria** | | | | | | | | | | | | | | | | | | | | |
| | Women | 0 | 12618 | 7479 | 34026 | **54123** | 0 | 14275 | 8903 | 40729 | **63907** | 0 | 15076 | 10211 | 50183 | **75470** | 0 | 15459 | 10937 | 59572 | **85968** |
| | Men | 1812 | 3212 | 10378 | 16576 | **31978** | 1992 | 3606 | 12754 | 20915 | **39267** | 2059 | 3714 | 14354 | 26088 | **46215** | 1671 | 3752 | 15258 | 30293 | **50974** |
| | Total | 1812 | 15830 | 17857 | 50602 | **86101** | 1992 | 17881 | 21657 | 61644 | **103174** | 2059 | 18790 | 24565 | 76271 | **121685** | 1671 | 19211 | 26195 | 89865 | **136942** |

y = years; [a] Population estimates from 2020 to 2080 retrieved from INE (2021), https://ine.pt/xportal/xmain?xpid=INE&xpgid=ine_indicadores&indOcorrCod=0010035&contexto=bd&selTab=tab2

Suppl. Table C - Estimated population and number of people with dementia in Portugal for 2020 - 2080 for the low growth scenario of population (continuation)

| | | 2060 | | | | | 2070 | | | | | 2080 | | | | |
|---|---|---|---|---|---|---|---|---|---|---|---|---|---|---|---|---|
| | | **65-69y** | **70-74y** | **75–79y** | **≥80 y** | **Total** | **65–69y** | **70–74y** | **75–79y** | **≥80 y** | **Total** | **65–69y** | **70–74y** | **75–79y** | **≥80 y** | **Total** |
| **Population**[a] | | | | | | | | | | | | | | | | |
| | Women | 278936 | 284259 | 313450 | 859439 | **1736084** | 259224 | 275579 | 266010 | 790273 | **1591086** | 209295 | 236043 | 249736 | 731331 | **1426405** |
| | Men | 226460 | 223178 | 229928 | 497211 | **1176777** | 214810 | 220792 | 200972 | 450367 | **1086941** | 169523 | 187740 | 194403 | 434968 | **986634** |
| | Total | 505396 | 507437 | 543378 | 1356650 | **2912861** | 474034 | 496371 | 466982 | 1240640 | **2678027** | 378818 | 423783 | 444139 | 1166299 | **2413039** |
| **Dementia cases according** | **10/66 DRG criteria** | | | | | | | | | | | | | | | |
| | Women | 1395 | 18818 | 30812 | 168536 | **219561** | 1296 | 18243 | 26149 | 154973 | **200661** | 1046 | 15626 | 24549 | 143414 | **184635** |
| | Men | 5729 | 12967 | 25384 | 91338 | **135418** | 5435 | 12828 | 22187 | 82732 | **123182** | 4289 | 10908 | 21462 | 79904 | **116563** |
| | Total | 7124 | 31785 | 56196 | 259874 | **354979** | 6731 | 31071 | 48336 | 237705 | **323843** | 5335 | 26534 | 46011 | 223318 | **301198** |
| | **DSM-IV criteria** | | | | | | | | | | | | | | | |
| | Women | 0 | 11598 | 9059 | 67380 | **88037** | 0 | 11244 | 7688 | 61957 | **80889** | 0 | 9631 | 7217 | 57336 | **74184** |
| | Men | 1427 | 2879 | 12692 | 33810 | **50808** | 1353 | 2848 | 11094 | 30625 | **45920** | 1068 | 2422 | 10731 | 29578 | **43799** |
| | Total | 1427 | 14477 | 21751 | 101190 | **138845** | 1353 | 14092 | 18782 | 92582 | **126809** | 1068 | 12053 | 17948 | 86914 | **117983** |

y = years; [a] Population estimates from 2020 to 2080 retrieved from INE (2021), https://ine.pt/xportal/xmain?xpid=INE&xpgid=ine_indicadores&indOcorrCod=0010035&contexto=bd&selTab=tab2
